# A case of laparotomic cholecystectomy in a patient with biventricular assist devices

**DOI:** 10.1186/s40981-017-0078-5

**Published:** 2017-02-07

**Authors:** Kenta Okitsu, Takeshi Iritakenishi, Chiyo Ootaki, Yuji Fujino

**Affiliations:** 1 Branch of Anesthesiology and Intensive Care Medicine, Osaka University Graduate School of Medicine, Osaka, Japan; 2 2-2 Yamadaoka, Suita, Osaka, 565-0871 Japan

**Keywords:** Biventricular assist devices, Non-cardiac surgery, Near infrared spectroscopy, Transesophageal echocardiography

## Abstract

We describe a patient with biventricular assist devices who had systemic inflammation because of cholecystitis that required open cholecystectomy, and we discuss the anesthetics and monitors that should be used in unstable patients with ventricular assist devices (VADs) who are undergoing major surgery.

The patient was a 40-year-old man in the dilated phase of hypertrophic obstructive cardiomyopathy, who was implanted with an internal left VAD and external right VAD. We anesthetized the patient with a combination of a low dose of sevoflurane and ketamine to minimize vasodilation. We chose ketamine because we expected it to have a postoperative analgesic effect. An INVOS™ (Medtronic) monitor was beneficial, especially since the pulse oximeter did not work because of a pulse deficit. The FloTrach™ (Edwards Lifesciences) failed to measure the stroke volume and its variability. The left VAD, the Jarvik2000, did not show its flow rate. However, we were able to estimate that the flow was stabilized, because the flow rate of the right VAD was stable, and there was no significant change in both ventricles and septa, as shown on transesophageal echocardiography.

## Background

Ventricular assist devices (VADs) are increasingly indicated in patients with acute or chronic heart failure refractory to medication. They are used as a temporary mechanical assist, permanent therapy, or bridging therapy for heart transplantation. It is common for physicians to manage anesthetic drugs in patients with VADs who undergo non-cardiac surgeries or procedures that require sedation such as gastrointestinal endoscopy. Successful management with non-invasive monitors has been reported in patients who have relatively stable hemodynamics [1–3]; however, managing unstable patients who are undergoing major surgery is challenging. We describe a patient with systemic inflammation and biventricular assist devices (BiVADs) who underwent laparotomic cholecystectomy.

## Case presentation

Written informed consent was obtained from the patient to publish this case report. A 40-year-old male patient with BiVAD support required cholecystectomy because of cholecystitis that could not be controlled with internal medication. He was diagnosed as having hypertrophic obstructive cardiomyopathy (HOCM) at the age of 25. His heart function became increasingly worse after HOCM progressed to the dilated phase. Severe heart failure occurred when he was 39 years old, and his circulation and oxygenation were insufficient even with medication, intra-aortic balloon pumping, and percutaneous cardiopulmonary support. Surgical aortic valve replacement, tricuspid valve plasty, and external left VAD (LVAD) installation were performed; however, he did not recover and right heart failure advanced. An external right VAD was required, and the LVAD was converted to an implanted one (Jarvik2000, Jarvik Heart, Inc., New York, NY) while the patient waited for heart transplantation. During these treatments for heart failure, he developed non-occlusive mesenteric ischemia and ileus, which required open abdominal surgery four times under general anesthesia. Soon after BiVAD surgery, he developed cholecystitis. Percutaneous transhepatic gallbladder drainage and antibiotics were continued for 1 month, but these treatments were ineffective. Cholecystectomy with laparotomy was planned because laparoscopic surgery was considered difficult, as the pipelines and drivelines of the VADs would prevent the use of an appropriate approach, and we expected strong adhesion caused by previous laparotomies.

He had been managed in an intensive care unit (ICU) for more than 200 days before the surgery. An arterial catheter and central venous catheter were placed preoperatively. He was not sedated and spontaneously ventilated, although tracheostomy had been performed, and supplemental oxygen was required because he had not recovered from a BiVAD procedure-related lung injury. No inotrope or vasoconstrictor was used preoperatively. Warfarin was discontinued 5 days before the surgery, and intravenous unfractionated heparin was administered until 6 h preoperatively. The international normalized ratio of prothrombin time (PT-INR) was 1.2 just before the patient entered the operating theater. General anesthesia was induced and maintained with a low dose of sevoflurane inhalation (0.8 to 1.0% end-tidal concentration) combined with a sub-anesthetic, intravenous dose of ketamine (30 mg bolus administration followed by a 0.5 mg/kg/h continuous infusion). Muscular relaxation was obtained using rocronium, and the patient was mechanically ventilated throughout the operation. Pressure control ventilation was chosen with the peak inspiratory pressure 20 cmH_2_O, respiratory rate from 10 to 12 times per minute and positive end-expiratory pressure 4 cmH_2_O. In addition to the basic monitors, we placed an INVOS™ (Medtronic, Minneapolis, MN) on the patient’s forehead to detect cerebral tissue oxygenation and on the foot to determine peripheral tissue oxygenation. We performed transesophageal echocardiography (TEE) using a pediatric probe to avoid esophageal injury, as a 14-French nasogastric tube was placed preoperatively, and the surgeons requested us to maintain it intraoperatively. We converted the arterial pressure measurement kit to the FloTrach™ (Edwards Lifesciences, Irvine, CA) to test its feasibility, although it could not estimate the stroke volume because of the low amplitude of waveform. The Jarvik2000 did not show the flow rate, so we estimated that the flow was stabilized because TEE did not show any significant change in the size and shape of both ventricles and septa, and the external right VAD flow rate was maintained at 3.8–3.9 L/min throughout the procedure.

Intraoperative analgesia was obtained with ketamine, and a 0.1 μg/kg/min infusion of remifentanil. 300 μg of fentanyl was administered to achieve the predicted target blood concentration 1 μg/ml at the time of postoperative ICU admission.

**Fig. 1:**
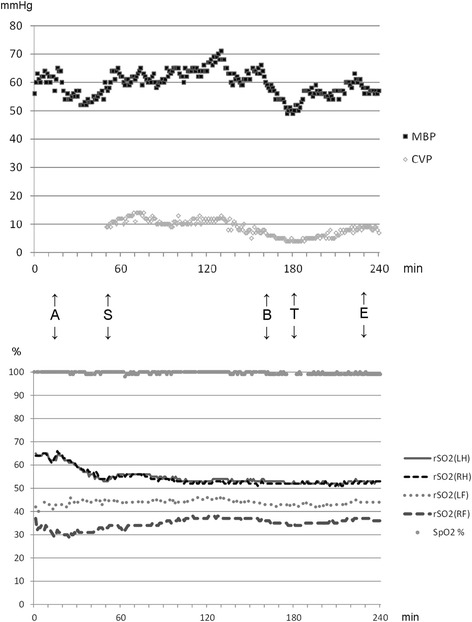
Trends in the perioperative mean blood pressure, central venous pressure, oxygen saturation of the pulse oximeter, and regional saturation of oxygen. *MBP* mean blood pressure, *CVP* central venous pressure, *rSO2* regional saturation of oxygen, *LH* left head, *RH* right head, *LF* left foot, *RF* right foot, *SpO2* oxygen saturation of the pulse oximeter, *A* start of anesthesia, *S* start of surgery, *B* bleeding, *T* transfusion, *E* end of surgery

The surgeons had difficulty separating the adhesion and achieving hemostasis. When the blood loss exceeded 300 mL, we observed a moderate decrease in the mean blood pressure (from 60 to 50 mmHg) and central venous pressure (from 7 to 5 cmH_2_O), and a temporary decrease in the pulse oximetry value (Fig. 1). However, there was no decrease in the flow of the VADs, and TEE showed no change in the right and left ventricular sizes. We decided to transfuse two units of packed red blood cells when the blood loss reached 400 mL, because the patient had preoperative anemia. After transfusion, the blood pressure, central venous pressure, and pulse oximetry values restored. The surgery was successfully finished. The durations of surgery and anesthesia were 191 and 270 min, respectively. The total volume of infusion, bleeding, and urine were 810, 420, 45 mL, respectively. We did not use any inotropes or vasoconstrictors. We performed a transversus abdominis plane block using a posterior approach postoperatively. We awoke the patient in the operating theater, and his spontaneous ventilation recovered. Postoperative adverse events associated with anesthetic management were not observed. Postoperative analgesic effect of intraoperative low dose ketamine and transversus abdominis plane block was unclear because the patient was slightly sedated using dexmedetomidine during the first postoperative 24 h in the ICU, and we could not assess the pain scale. Cholecystitis was successfully treated, although he required furthermore ICU stay to manage hemodynamics and wound infection around the LVAD driveline exit cite. He underwent heart transplantation after a 120-day waiting duration from the cholecystectomy.

## Discussion

We successfully performed anesthetic management in a patient with BiVAD support who underwent laparotomic cholecystectomy. The type of anesthetic agent, the decision of transfusion, and invasive monitors we used are important parts of our management.

Various anesthetic agents have been used during non-cardiac surgery in patients with LVAD, although no specific one has been recommended yet [4, 5]. We used ketamine combined with a low dose of sevoflurane, because ketamine causes sympathetic stimulation [6] and can be a good choice for hemodynamically unstable patients [7]. We also chose ketamine because of its postoperative analgesic effect, which has been previously reported [8, 9]. Other possible agents we could have used to minimize a negative hemodynamic effect include midazolam, dexmedetomidine, and etomidate. We did not use midazolam, because it had been administered to the patient over a long duration in the ICU, and we were informed that the patient might have developed a tolerance to it. We did not use dexmedetomidine, because using this agent for anesthetic maintenance is considered off-label use in our country. Regarding etomidate, it is not available in our country.

We transfused red blood cells even though blood loss was insignificant. In previous reports on patients with LVAD who underwent cholecystectomy, blood transfusion was necessary in approximately half of the cases [10, 11]. We decided to transfuse blood despite the preserved hemodynamic parameters because the patient had preoperative anemia, and there was the possibility of early postoperative bleeding. The blood oozing did not completely stop even though a large amount of oxidized cellulose was used. We should have considered this issue with point-of-care transfusion; however, point-of-care coagulation and platelet function testing devices were not available at that time.

In patients with continuous flow VADs, obtaining a non-invasive blood pressure measurement by using the automated cuff and pulse oximeter is sometimes unreliable because of the narrow pulse pressure. Near infrared spectroscopy may be feasible in such patients [12]. When we observed a temporal pulse-wave deficit and the pulse oximeter did not work well, the INVOS™ provided us with important information about the central and peripheral oxygenation. We also used it to detect the possibility of cerebral infarction. Warfarin was discontinued to reduce the risk of perioperative bleeding, and the PT-INR was maintained around 1.2. It is important to detect cerebral thrombosis as soon as possible, because it is associated with a high mortality rate in patients with VADs [13].

Concerning invasive monitors, arterial and central venous catheters were already placed in our patient and we only added TEE. Stone et al. reported that the opportunity to use an invasive arterial pressure monitoring catheter and central venous access catheter was decreasing, and TEE was only used in less than 15% of 291 non-cardiac procedures performed over 10 years at their institution [1]. However, the use of TEE was important because the Jarvik2000 does not show its flow rate.

The intraoperative flow of the right ventricular assist device was stable, and TEE showed that there was no significant change in the size and shape of both ventricles, and no shifting of the septa. Thus, we were able to estimate that the flow of the LVAD was also stable. Moreover, we could detect the mechanical obstruction of pipelines of the VAD by TEE. However, successful imaging is not promised when a gastric tube is placed simultaneously. Moreover, the risk of esophageal injury may be increased even though we use a pediatric probe. We also should carefully consider the risk of bleeding because patients with VADs may have a coagulation abnormality. Additionally, the risk and benefit of TEE should be considered in each case before it is used.

## Conclusions

We successfully managed the anesthetics of a patient with BiVADs who underwent open cholecystectomy. Anesthesia and analgesia were achieved with sevoflurane, ketamine, remifentanil, fentanyl, and a TAP block under stable hemodynamic parameters without any dysfunction of the VADs. INVOS™ was beneficial, whereas FloTrach™ was not. TEE was also useful, although its indication should be carefully considered.

## References

[1] Stone M, Hinchey J, Sattler C, Evans A (2016). Trends in the management of patients with left ventricular assist devices presenting for noncardiac surgery: a 10-year institutional experience. Semin Cardiothorac Vasc Anesth.

[2] Nelson EW, Heinke T, Finley A, Guldan GJ, Gaddy P, Matthew Toole J, Mims R, Abernathy JH (2015). Management of LVAD patients for noncardiac surgery: a single-institution study. J Cardiothorac Vasc Anesth.

[3] Goudra BG, Singh PM (2013). Anesthesia for gastrointestinal endoscopy in patients with left ventricular assist devices: initial experience with 68 procedures. Ann Card Anaesth.

[4] Stone ME, Soong W, Krol M, Reich DL (2002). Anesthetic considerations in patients with ventricular assist devices presenting for non-cardiac surgery. Anesth Analg.

[5] Kocabas S, Askar FZ, Yagdi T, Engin C, Ozbaran M (2013). Anesthesia for ventricular assist device placement: experience from a single center. Transplant Proc.

[6] Pandit JJ (2008). Intravenous anaesthetic agents. Anaesth Intensive Care Med.

[7] Morris C, Perris A, Klein J, Mahoney P (2009). Anaesthesia in haemodynamically compromised emergency patients: does ketamine represent the best choice of induction agent?. Anaesthesia.

[8] Bell RF, Dahl JB, Moore RA, Kalso EA (2006). Perioperative ketamine for acute postoperative pain. Cochrane Database Syst Rev.

[9] Jouguelet-Lacoste J, La Colla L, Schilling D, Chelly EC (2015). The use of intravenous infusion or single dose of low-dose ketamine for postoperative analgesia: a review of the current literature. Pain Med.

[10] Morgan JA, Paone G, Nemeh HW, Henry SE, Gerlach B, Williams CT, Lanfear DE, Tita C, Brewer RJ (2012). Non-cardiac surgery in patients on long-term left ventricular assist device support. J Heart Lung Transplant.

[11] Garatti A, Bruschi G, Colombo T, Russo C, Milazzo F, Catena E, Lanfranconi M, Vitali E (2009). Noncardiac surgical procedures in patient supported with long-term implantable left ventricular assist device. Am J Surg.

[12] Maldonado Y, Singh S, Taylor MA (2014). Cerebral near-infrared spectroscopy in perioperative management of left ventricular assist device and extracorporeal membrane oxygenation patients. Curr Opin Anesthesiol.

[13] Jennings DL, Jacob M, Chopra A, Nemerovski CW, Morgan JA, Lanfear DE (2014). Safety of anticoagulation reversal in patients supported with continuous-flow left ventricular assist devices. ASAIO J.

